# Small interfering RNA library screen identified polo-like kinase-1 (PLK1) as a potential therapeutic target for breast cancer that uniquely eliminates tumor-initiating cells

**DOI:** 10.1186/bcr3107

**Published:** 2012-02-06

**Authors:** Kaiji Hu, Jennifer H Law, Abbas Fotovati, Sandra E Dunn

**Affiliations:** 1 Laboratory for Oncogenomic Research, Departments of Pediatrics, Experimental Medicine, and Medical Genetics, Child and Family Research Institute, University of British Columbia, 950 W. 28th Ave, Vancouver, British Columbia, V5Z 4H4, Canada

## Abstract

**Introduction:**

Triple-negative breast cancer (TNBC) high rate of relapse is thought to be due to the presence of tumor-initiating cells (TICs), molecularly defined as being CD44^high^/CD24^-/low^. TICs are resilient to chemotherapy and radiation. However, no currently accepted molecular target exists against TNBC and, moreover, TICs. Therefore, we sought the identification of kinase targets that inhibit TNBC growth and eliminate TICs.

**Methods:**

A genome-wide human kinase small interfering RNA (siRNA) library (691 kinases) was screened against the TNBC cell line SUM149 for growth inhibition. Selected siRNAs were then tested on four different breast cancer cell lines to confirm the spectrum of activity. Their effect on the CD44^high ^subpopulation and sorted CD44^high^/CD24^-/low ^cells of SUM149 also was studied. Further studies were focused on polo-like kinase 1 (PLK1), including its expression in breast cancer cell lines, effect on the CD44^high^/CD24^-/low ^TIC subpopulation, growth inhibition, mammosphere formation, and apoptosis, as well as the activity of the PLK1 inhibitor, BI 2536.

**Results:**

Of the 85 kinases identified in the screen, 28 of them were further silenced by siRNAs on MDA-MB-231 (TNBC), BT474-M1 (ER^+^/HER2^+^, a metastatic variant), and HR5 (ER^+^/HER2^+^, a trastuzumab-resistant model) cells and showed a broad spectrum of growth inhibition. Importantly, 12 of 28 kinases also reduced the CD44^high ^subpopulation compared with control in SUM149. Further tests of these 12 kinases directly on a sorted CD44^high^/CD24^-/low ^TIC subpopulation of SUM149 cells confirmed their effect. Blocking PLK1 had the greatest growth inhibition on breast cancer cells and TICs by about 80% to 90% after 72 hours. PLK1 was universally expressed in breast cancer cell lines, representing all of the breast cancer subtypes, and was positively correlated to CD44. The PLK1 inhibitor BI 2536 showed similar effects on growth, mammosphere formation, and apoptosis as did PLK1 siRNAs. Finally, whereas paclitaxel, doxorubicin, and 5-fluorouracil enriched the CD44^high^/CD24^-/low ^population compared with control in SUM149, subsequent treatment with BI 2536 killed the emergent population, suggesting that it could potentially be used to prevent relapse.

**Conclusion:**

Inhibiting PLK1 with siRNA or BI 2536 blocked growth of TNBCs including the CD44^high^/CD24^-/low ^TIC subpopulation and mammosphere formation. Thus, PLK1 could be a potential therapeutic target for the treatment of TNBC as well as other subtypes of breast cancer.

## Introduction

Triple-negative breast cancer (TNBC) is considered the most aggressive breast cancer subtype because it is associated with the greatest probability of early relapse and death [1-3]. It is estimated that more than 1 million women are diagnosed with breast cancer annually, and TNBC accounts for about 15% of those cases [4]. They are challenging clinically for a number of reasons. They do not express the estrogen receptor (ER), progesterone receptor, and human epidermal growth factor 2 (HER2). Therefore, patients are not candidates for targeted agents, such as antiestrogens and trastuzumab, that afford the greatest survival benefit for eligible patients. The prognosis for patients with this type of tumor is very poor, not only because hormonal therapy and treatment with trastuzumab are inapplicable, but also because these tumors seem to be more aggressive than other breast cancer subtypes [5].

Although it is highly sensitive to chemotherapy, the progression-free time of TNBC, however, is generally short, and has greater recurrence rates than those of non-TNBC tumors during the first and third years after their initial diagnosis, as well as a higher 5-year mortality rate [3,4]. The high rates of early relapse indicate that the tumor cells rapidly adapt to the insult of chemotherapy by inducing resistance mechanisms. In addition, the adverse side effects of traditional chemotherapy are inevitable for patients with TNBC, which leads to the notable morbidity associated with treating this particular breast cancer subtype. Thus, identifying specific molecular targets against TNBC is timely and essential.

No currently accepted therapeutic target is known for TNBC, unlike some other subtypes of breast cancer [4]. ER-expressing breast tumors, for instance, can be treated with tamoxifen and aromatase inhibitors, and HER2-expressing ones can be treated with trastuzumab. Ongoing studies are searching for new drug targets against TNBC. One such development is the inhibition of poly (ADP-ribose)-polymerase 1 (PARP1) [4,6]. PARP1 plays a vital role in repairing DNA damage together with other mechanisms that involve *BRCA1 *and *BRCA2*. The combination of the mutation of *BRCA *and PARP inhibition attributed to so-called synthetic lethality [6,7]. The impressive clinical phase II results involving these criteria have led to a definitive phase III study [4]. Although this is promising, *BRCA1 *and *BRCA2 *mutations account for slightly more than 10% of breast cancers that are triple-negative [8]. Other therapeutic targets under development for TNBC include epidermal growth factor receptor (*EGFR*), mammalian target of rapamycin (*mTOR*), the RAS-mitogen-activated protein kinase signaling pathway (*Raf*/*Mek*/*MAP*), and Src tyrosine kinase [4,9]. However, some of these proposed targets are applicable only in more-specific subgroups of TNBC, and the ways to tackle the tumor-initiating subpopulation, which is believed to be the root cause of the relapse of cancer, have not been fully studied. For breast cancer, it has been proposed that the subpopulation cells of CD44^high^/CD24^-/low ^have cancer stem cell properties [10,11]. Such cancer stem cells or tumor-initiating cells (TICs) are resistant to traditional chemotherapies and are considered to be responsible for cancer relapse [10-13]. It has been reported that treatment with traditional chemotherapies leads to enriched TICs both *in vitro *and *in vivo *[14-17]. Thus targeting the bulk cancer cell population, as well as TICs, should be considered at the early stage of the search for therapeutic targets.

Kinases play an essential role in the processes of protein phosphorylation and are deregulated in many diseases, such as cancer. Numerous studies have proved that many kinases are critical in cancer cell survival under both *in vitro *and *in vivo *conditions [18-20]. Kinases are eminently the most treatable with drugs. Some new drugs of kinase inhibitor, such as imatinib (Gleevec), fasudil, and rapamycin, have been successfully developed and applied clinically for treatment of a variety of cancers [21,22]. For TNBC, it has been shown that several kinases could be targeted for growth inhibition, including *MAP *kinase, Src tyrosine kinase (*PDGFR, EGFR, IGF-1R*, and *HGFR*), *RSK *kinases [4,9,23,24]. More important, targeting kinases resulting in growth inhibition of TICs of different cancers has been reported [25,26]. Prochownik *et al. *[13,27] found that CGP74514A and rottlerin, which are kinase inhibitors of CDK1/cyclin B and PKC, respectively, can selectively inhibit cancer stem cells isolated from the breast cancer cell line MCF7. The availability of a large kinase small interfering RNA (siRNA) library provides an excellent tool for an unbiased genome-wide screen for active kinases as potential therapeutic targets against not only the bulk cancer cells but also TICs.

In this study, we first performed a genome-wide human kinase siRNA library screen against a TNBC cell line SUM149 for growth inhibition. A panel of selected active kinases was then further tested on four different breast cancer cell lines to confirm the spectrum of growth inhibition. Several kinases that also inhibited the tumor-initiating CD44^high ^population in SUM149 after siRNA treatments were identified and tested directly against sorted CD44^high^/CD24^-/low ^cells of SUM149. The most impressive kinase lead was polo-like kinase 1 (*PLK1*). Therefore, we focused on *PLK1 *inhibition as the best potential therapeutic lead for TNBC by showing that it is highly expressed in breast cancer cell lines, and its inhibition by *PLK1 *siRNA as well as BI 2536, an ATP-competitive inhibitor designed to inhibit PLK1 [28], killed the CD44^high^/CD24^-/low ^population and induced apoptosis. Combined treatment with drugs and BI 2536 greatly inhibited the growth of TNBC. Therefore, it offers potential as a better therapeutic target for TNBC.

## Materials and methods

### Cell culture

SUM149 cells were purchased from Astrand (Ann Arbor, MI, USA) and cultured in F-12 (Ham) media (Gibco/Invitrogen, Burlington, ON, Canada) supplemented with 5 μg/ml insulin (Sigma-Aldrich, Oakville, ON, Canada), 1 μg/ml hydrocortisone (Sigma-Aldrich), 10 m*M *HEPES (Sigma-Aldrich), and 5% fetal bovine serum (FBS; Gibco/Invitrogen). MDA-MB-231 and MCF7 were purchased from ATCC and cultured in Dulbecco Modified Eagle medium (DMEM, Gibco/Invitrogen) with 10% FBS. BT474-M1, a metastatic variant of BT474, was a gift of Dr. Mien-Chie Hung (MD Anderson Cancer Center, Houston, TX, USA). HR5, which is derived from BT474 and is resistant to trastuzumab, was from Dr. Carlos Arteaga (Vanderbilt-Ingram Cancer Center, Nashville, TN, USA). They were both cultured in DMEM-F12 (1:1) with 10% FBS. AU565, HCC1937, and T47D (ATCC) were cultured in RPMI-1640 media supplemented with 5% FBS (Gibco/Invitrogen), 10 m*M *HEPES, 4.5 g/L glucose, 1 m*M *sodium pyruvate, and 100 units/ml penicillin/streptomycin (Sigma-Aldrich). All the cells were incubated at 37°C with 5% CO_2_, and subcultured twice weekly during the experimental period.

### Kinase siRNA library

The siRNA library (Vision 2) of 691 human kinases was purchased from Qiagen (Toronto, ON, Canada). Two different sequences of siRNA target each of the genes in the library. The siRNA stock samples were diluted to working stocks at 2 μ*M *on arrival by following the manufacturer's instructions and stored at -20°C before use.

### Kinase siRNA library screen

The screening methods were previously described [19]. In brief, SUM149 cells were seeded (5,000 cells/well) into 96-well plates (BD; Becton Dickinson, Franklin Lakes, NJ, USA) overnight. The cells were transfected with siRNA in Lipofectamine RNAiMAX (Invitrogen) at 5 n*M *for 72 hours. Cells were then fixed in 2% paraformaldehyde (Sigma-Aldrich) with nuclear dye, Hoechst 33342 (1 μg/ml) (Sigma-Aldrich). After a gentle wash with phosphate-buffered solution (PBS), the cells were kept in fresh PBS, and the plates were kept at 4°C in the dark before analysis on the ArrayScan high-content screening system (HCS; Thermo Fisher Scientific, Pittsburgh, PA, USA). Twenty view fields per well were scanned and analyzed. The screen was repeated once to confirm the activity of siRNAs. Cells treated with Lipofectamine RNAiMAX alone without siRNA served as controls. Additionally, scrambled siRNAs and green fluorescent protein siRNAs, which were included in the library, served as internal references in each assay plate. Apoptosis was identified by nuclear morphology and Hoechst dye intensity by the HCS system [19], which allows simultaneously acquiring quantitative cellular data and images of each individual cell sample. Growth inhibition was calculated as a percentage of the control. To focus on the most important kinases, only those siRNAs that were active for both sequences and showed a minimum of 30% inhibition compared with control were considered to be active in the screen.

### Effect of the active kinases on the growth of different breast cancer cell lines

A panel of 28 active kinases was selected from the hit list, based on their activity and classes, and silenced by their corresponding siRNAs in four breast cancer cell lines, MDA-MB-231, SUM149, BT474-M1, and HR5. Cell lines MDA-MB-231 and SUM149 are TNBC, whereas the latter two are HER2 positive. Unless otherwise stated, all growth assays in the study were done in replicates of three or five and repeated at least once to confirm the activity.

### Effect of the selected kinases on CD44 ^high ^subpopulation of SUM149

SUM149 cells were treated with the selected siRNAs at 5 n*M*, as described in a previous section. After 72 hours of treatment, the cells were fixed in 2% paraformaldehyde with nuclear dye, Hoechst 33342, at room temperature for 30 minutes. The cells were then washed gently 3 times with PBS and stained with 40 μl/well of mouse anti-human CD44-PE conjugated antibody (BD Biosciences, Mississauga, ON, Canada; 1:100 dilution) at room temperature for 1 hour in the dark. The samples were then washed with PBS and kept at 4°C in the dark before analysis with the HCS system for the CD44^high ^cells surviving the siRNA treatments.

### Effect of the selected kinases on sorted CD44^high^/CD24^-/low ^TIC subpopulation of SUM149

SUM149 cells were cultured and sorted for the CD44^high^/CD24^/-low ^subpopulation as described [14] to test directly the effect of the active kinases on TICs. Sorted cells were seeded at 5,000 cells/well into 96-well culture plates (BD) and cultured overnight. The siRNAs of the 12 selected kinases were then added as described earlier. Cells treated with Lipofectamine RNAiMAX alone without siRNA served as controls. Additionally, scrambled siRNAs were included in the experiments, and served as internal reference in each assay plate. The treatment lasted for 72 hours. The treated cells were then fixed and stained with Hoechst dye, and the growth inhibition was analyzed with the HCS system, as described in previous sections.

### PLK1 expression in different breast cancer cell lines and its correlation to CD44

PLK1 protein expression in eight breast cancer cell lines, SUM149, MDA-MB-231, BT474, HR5, HR6, MCF7, HCC1937, and AU565, was investigated with Western blot, as previously described [29]. In brief, proteins were isolated from log-phase growing cells of these six cell lines by using an ELB buffer [24]. PLK1 (Abcam, Cambridge, MA, USA; 1:2,000 dilution) and actin (Cell Signaling, Pickering, ON, Canada; 1:5,000 dilution) were detected with immunoblotting.

To confirm the silencing efficacy of *PLK1 *siRNA on PLK1 expression, SUM149 and MBA-MB-231 were seeded into six-well culture plates (BD) at 350,000 cells/well in 2 ml corresponding media. *PLK1 *and control siRNAs were added to achieve 5 n*M *final concentration, and Lipofectamine RNAiMAX alone without siRNA served as the control. The sample plate was then incubated for 72 hours. After harvesting the cells and extracting the proteins, PLK1 expression was detected with immunoblotting (1:2,000 for PLK1 and 1:5,000 for actin), as described earlier.

To explore the possible connection between PLK1 and CD44, SUM149 cells were seeded onto eight-chamber slides (BD), washed with PBS, fixed with 2% formaldehyde for 20 minutes, rinsed twice with PBS, and then incubated with PBS containing 0.1% Triton X-100 (Sigma-Aldrich) for 30 minutes. Next, the slides were washed with PBS and incubated with mouse anti-CD44 (BD Biosciences; 1:200 dilution) and rabbit anti-PLK1 (LifeSpan Bioscience Inc., Seattle, WA, USA; 1:400 dilution) antibodies diluted in buffer containing 10% bovine serum albumin and 2% goat serum overnight at 4°C in a humidified container. After washing 3 times with PBS, glass slides were incubated with Alexa Fluor 546 anti-mouse and Alexa Fluor 488 anti-rabbit antibodies (Invitrogen; 1:1,000 dilution) for 1 hour, washed 3 times, and then mounted by using Prolong Gold (Invitrogen) with 4ʹ,6-diamidino-2-phenylindole (DAPI; Invitrogen). Cells were observed with a Zeiss AX10 microscope and photographed by using an Olympus DP72 digital camera. All cells in three randomly selected view fields (×10 magnification) were surveyed for CD44 and PLK1 expression, and the percentage of CD44^high ^cells that were also PLK1^high ^was calculated.

### PLK1 activity after inhibition by BI 2536 (a known PLK1 small-molecular inhibitor)

The effect of PLK1 inhibitor on PLK1 activity was studied with an immunofluorescence method. SUM149 cells were seeded on glass coverslips in six-well dishes and treated with dimethyl sulfoxide (DMSO) or BI 2536 at 25 n*M *or 100 n*M *for 72 hours. Fixed cells were then stained with rabbit anti-phospho-cyclin B1 (S133) (Cell Signaling; 1:200 dilution), which is a known downstream substrate of PLK1 [26]. This was followed by secondary antibody and image acquisition, as described earlier.

For quantitative analysis of PLK1 activity, SUM149 cells were seeded at 3,000 cells/well overnight and treated with DMSO or BI 2536 at 10 to 100 n*M *in 96-well plates for 72 hours. Fixed cells were then stained with the cyclin B1 antibody, as described earlier, except that Hoechst was used, and the cells were kept in PBS before analyzing with the HCS system.

### Growth inhibition of BI 2536 on different breast cancer cells and TICs

Prior studies reported that BI 2536 is highly selective for PLK1 when tested against 1,000 related kinases [28]. BI 2536 (Sigma-Aldrich) was prepared in DMSO and tested against seven cell lines, SUM149, MDA-MB-231, BT474-M1, HR5, MCF7, AU565, and T47D. Each cell line was seeded at 3,000 cells/well and incubated overnight. Cells were then treated with BI 2536 at concentrations of 1 to 100 n*M *in the medium for 72 hours. Propidium iodide (PI, Sigma-Aldrich) and Hoechst dye solution were added 40 minutes before the end of treatments to each well at a final concentration of 1 μg/ml for each dye. The sample plates were then scanned live with the HCS system. Growth inhibition was calculated as a percentage of the control without the DMSO and the drug, and the samples treated with DMSO alone served as a reference. To address whether a longer period of treatment would increase the efficacy of the drug compound, SUM149 cells were treated with BI 2536 for 10 days. The methods were the same as stated earlier, except that the seeding density was only 1,000 cells/well, and the media with BI 2536 were later replaced with fresh media containing BI 2536 at days 4 and 7 of the treatments.

To determine whether BI 2536 has a similar inhibitory effect on TICs as do the *PLK1 *siRNAs, sorted CD44^high^*/*CD24^-/low ^cells of SUM149 were seeded at a density of 3,000 cells/well in 96-well plates. They were then treated with BI 2536 at concentrations ranging from 1 to 100 n*M *for 72 hours.

Mammosphere assays were performed with SUM149, as well as with MDA-MB-231 cells, which highly expresses CD44 in about 90% of its population, in ultra-low attachment six-well culture plates (Corning, Lowell, MA, USA) in complete Mammocult media (StemCell Technologies, Vancouver, BC, Canada), as previously described [30]. DMSO control or BI 2536 (10 n*M *or 25 n*M*) was added at time of seeding (5,000 cells/well). Serial passaging was performed as per Subculture of Mammospheres protocol (StemCell Technologies). In brief, after 7 days in culture, mammospheres were counted, collected in a conical tube, and centrifuged at 350 *g *for 5 minutes. Pellets were triturated with trypsin-EDTA (Invitrogen) to break up mammospheres to single cells. Cold PBS with 2% FBS was added, and cells were centrifuged at 350 *g *for 5 minutes. Pellets were resuspended in Mammocult media, and cell counts were performed. The mammosphere assay was reseeded by using the same cell densities and treatments as described earlier.

Chemotherapeutic drugs like paclitaxel (Taxol), doxorubicin (Dox), and 5-fluorouracil (5FU) had been reported to induce resistance of cancer cells, and to this is probably attributed their induction of TICs in the surviving population [14,15,31,32]. To determine whether drug treatment followed by BI 2536 could overcome the TICs, characterized as CD44^high^/CD24^-/low^, SUM149 cells were seeded at 1,000 cells/well in 96-well plates overnight. Taxol, Dox, or 5FU (Sigma-Aldrich) at different concentrations were then added the following day, and the plates were incubated for 72 hours. One of the plates was then fixed and stained for Hoechst, CD44 APC (BD Biosciences; 1:50 dilution) and CD24 FITC (BD Biosciences; 1:10 dilution) antibodies, as described earlier, and analyzed with an HCS system for growth and CD44^high^/CD24^-/low ^cells. The medium in the second plate was removed and washed once with fresh medium. Then the medium with BI 2536 at different concentrations was added to the plate and incubated for another 4 days. The plate was fixed and analyzed with HCS, as described.

### Detection of apoptosis caused by BI 2536 on different breast cancer cell lines

To investigate apoptosis caused by BI 2536 on breast cancer cells of SUM149, MDA-MB-231, BT474-M1, and HR5, the cells after drug treatment were stained with PI or phospho-H2AX for quantification of apoptosis [14,19]. In brief, PI and Hoechst were added to cell wells at a final concentration of 1 μg/ml each, 40 minutes before the end of the 72-hour treatments. The sample plates were then scanned live with the HCS system. For phospho-H2AX, which is an early indicator of apoptosis [14,19], treated cells were fixed with 2% paraformaldehyde and Hoechst dye for 30 minutes followed by permeabilization with Triton X-100 (Fisher Scientific, Nepean, ON, Canada) and blocking with bovine serum albumin (Sigma-Aldrich) [19]. They were then incubated with mouse anti-human phospho-H2AX (Abcom; 1:100 dilution) for 1 hour at room temperature. This was followed by rabbit anti-mouse Alexa Fluor 488 antibody (Invitrogen; 1:100 dilution). The cells were gently washed with PBS after each procedure. The sample plates were finally analyzed, and images were taken by the HCS system.

## Results

### The siRNA library screen identified active kinases that significantly inhibited the growth of TNBC cell line SUM149

In the initial screen, 85 of the 691 kinases in total were identified to be significantly growth inhibitory (> 30% growth inhibition) on SUM149 cells once they were silenced by 5 n*M *siRNAs for 72 hours under the experimental conditions (Table 1; Table 1 of Additional file 1). These active kinases (about 12.3% of the kinome library tested) comprised a wide range of classes and functional groups, indicating that the cancer cell growth could be regulated through multiple genes and pathways. Of significant importance are the cell cycle-related kinases, *MAP *kinases, and protein kinases, as many identified active kinases belong to these groups. The critical roles they played in SUM149 cell growth and the strong sensitivity to siRNA silencing indicate their potential as therapeutic targets for TNBC. *PLK1*, in particular, is one of the most active kinases identified in the screen. The growth inhibition on SUM149 is more than 80%, with significant apoptosis of the cells under the experimental conditions.

**Table 1:** Partial list of the active kinases identified in the siRNA library screen

Accession number	Symbol	Brief description	Growth inhibition (%)
NM-001786	*CDC2*	Cell-division cycle 2, G_1 _to S and G_2 _to M	68^a^
NM-033487	*CDC2L1*	Cell-division cycle 2-like 1 (PITSLRE proteins)	71
NM-001274	*CHEK1*	CHK1 checkpoint homolog (*S. pombe*)	61
NM-001896	*CSNK2A2*	Casein kinase 2, alpha prime polypeptide	62^a^
NM-001929	*DGUOK*	Deoxyguanosine kinase	61
NM-000162	*GCK*	Glucokinase (hexokinase 4, maturity-onset diabetes of the young 2)	62
NM-153273	*IHPK1*	Inositol hexaphosphate kinase 1	55
NM-001569	*IRAK1*	Interleukin-1 receptor-associated kinase 1	59
XM-498294	*LOC392265*	Similar to cell-division protein kinase 5 (tau protein kinase II catalytic subunit) (TPKII catalytic subunit) (serine/threonine-protein kinase PSSALRE)	67
NM-005922	*MAP3K4*	Mitogen-activated protein kinase kinase kinase 4	57
NM-015133	*MAPK8IP3*	Mitogen-activated protein kinase 8 interacting protein 3	56
NM-006206	*PDGFRA*	Platelet-derived growth factor receptor, alpha polypeptide	63
NM-006212	*PFKFB2*	6-Phosphofructo-2-kinase/fructose-2,6-biphosphatase 2	60
NM-012395	*PFTK1*	PFTAIRE protein kinase 1	53
NM-000294	*PHKG2*	Phosphorylase kinase, gamma 2 (testis)	65
NM-018323	*PI4K2B*	Phosphatidylinositol 4-kinase type-II beta	51^a^
NM-004570	*PIK3C2G*	Phosphoinositide-3-kinase, class 2, gamma polypeptide	69^a^
NM-002651	*PIK4CB*	Phosphatidylinositol 4-kinase, catalytic, beta polypeptide	56
NM-181805	*PKIG*	Protein kinase (cAMP-dependent, catalytic) inhibitor gamma	65
NM-002658	*PLAU*	Plasminogen activator, urokinase	57
NM-005030	*PLK1*	Polo-like kinase 1 (*Drosophila*)	86^a^
NM-006254	*PRKCD*	Protein kinase C, delta	60
NM-016457	*PRKD2*	Protein kinase D2	60
NM-021135	*RPS6KA2*	Ribosomal protein S6 kinase, 90 kDa, polypeptide 2	50
NM-005983	*SKP2*	S-phase kinase-associated protein 2 (p45)	61
NM-003318	*TTK*	TTK protein kinase	64
NM-031432	*UCK1*	Uridine-cytidine kinase 1	63
NM-006296	*VRK2*	Vaccinia-related kinase 2	55

^a^Apoptosis is at least 5% more than the control for both siRNA sequences of the kinase, as measured by HCS system, based on nuclear properties (morphology and Hoechst dye intensity) of the cells. Note: Growth inhibition resulted from siRNA silencing of SUM149 cells at 5 n*M *for 72 hours. Data shown here are the average results of both siRNA sequences of the kinase from two independent screens. For a complete list of active kinases identified in the siRNA library screen, refer to Additional file 1.

### The active kinases showed a broad spectrum of growth inhibition on different breast cancer cell lines

**Figure 1:**
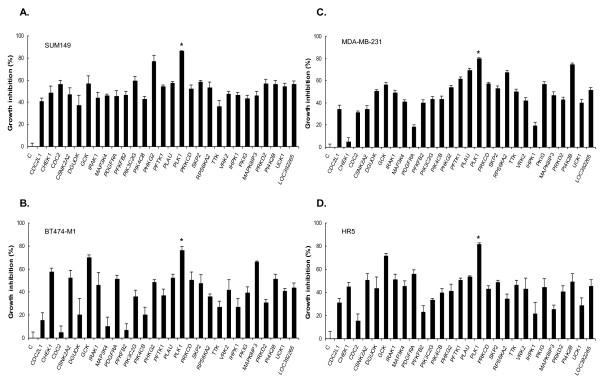
**Growth inhibition of the 28 active kinases on different breast cancer cells after siRNA silencing**. **(A) **Percentage growth inhibition of SUM149 by the kinases after siRNA silencing at 5 n*M *for 72 hours. C, control without siRNA. *PLK1. **(B) **Percentage growth inhibition of BT474-M1 by the kinases. **(C) **Percentage growth inhibition of MDA-MB-231 by the kinases. **(D) **Percentage growth inhibition of HR5 by the kinases. Data are presented as mean ± SD of two independent tests. Kinases in the figure: *CDC2L1*, cell-division cycle 2-like 1 (PITSLRE proteins); *CHEK1*, CHK1 checkpoint homologue (*S. pombe*); *CDC2*, cell-division cycle 2, G_1 _to S and G_2 _to M; *CSNK2A2*, casein kinase 2, alpha prime polypeptide; *DGUOK*, deoxyguanosine kinase; *GCK*, glucokinase (hexokinase 4, maturity-onset diabetes of the young 2); *IRAK1*, interleukin-1 receptor-associated kinase 1; *MAP3K4*, mitogen-activated protein kinase kinase kinase 4; *PDGFRA*, platelet-derived growth factor receptor, α polypeptide; *PFKFB2*, 6-phosphofructo-2-kinase/fructose-2,6-biphosphatase 2; *PIK3C2G*, phosphoinositide-3-kinase, class 2, γ polypeptide; *PIK4CB*, phosphatidylinositol 4-kinase, catalytic, β polypeptide; *PHKG2*, phosphorylase kinase, γ 2 (testis); *PFTK1*, PFTAIRE protein kinase 1; *PLAU*, plasminogen activator, urokinase; *PLK1*, polo-like kinase 1 (*Drosophila*); *PRKCD*, protein kinase C, delta; *SKP2*, S-phase kinase-associated protein 2 (p45); *RPS6KA2*, ribosomal protein S6 kinase, 90 kDa, polypeptide 2; *TTK*, TTK protein kinase; *VRK2*, vaccinia-related kinase 2; *IHPK1*, inositol hexaphosphate kinase 1; *PKIG*, protein kinase (cAMP-dependent, catalytic) inhibitor γ; *MAPK8IP3*, mitogen-activated protein kinase 8 interacting protein 3; *PRKD2*, protein kinase D2; *PI4K2B*, phosphatidylinositol 4-kinase type-II β; *UCK1*, uridine-cytidine kinase 1; *LOC392265*, similar to cell-division protein kinase 5 (Tau protein kinase II catalytic subunit) (TPKII catalytic subunit) (serine/threonine-protein kinase PSSALRE).

Although the initial kinase siRNA library screen was done on SUM149 cells, most of the 28 selected active kinases, once silenced by their corresponding siRNAs, showed a strong and broad spectrum of inhibitory effect on the growth of all four cell lines tested, SUM149, MDA-MB-231, BT474-M1, and HR5 (Figure 1). A few examples of such kinases are *PLK1, GCK, SKP2, PLAU, RPS6KA2, PI4K2B*, and *LOC392265*. In particular, these kinases are significantly active on HR5, a trastuzumab-resistant model. The results indicated that these kinases offer potential applications not only in TNBC but also in other subtypes of breast cancer.

### The active kinases reduced the CD44^high ^subpopulation and inhibited the growth of sorted CD44^high^/CD24^-/low ^cells of SUM149 after siRNA knockdown

**Figure 2:**
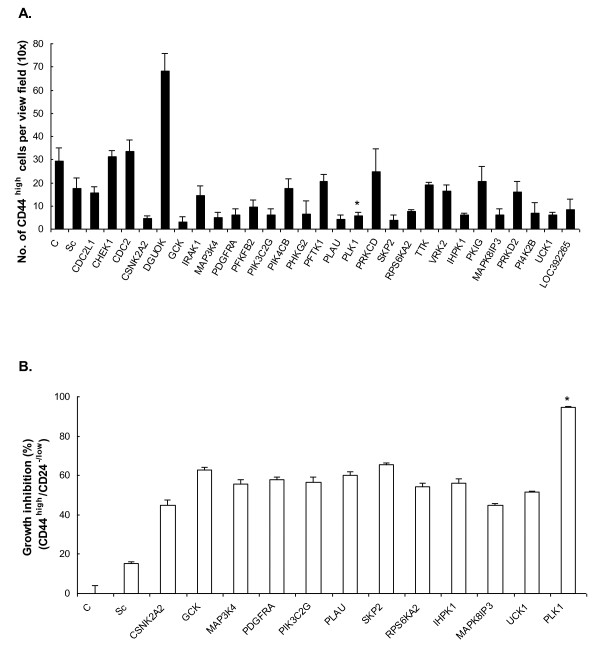
**Effect of the active kinases on CD44^high ^and the sorted CD44^high^/CD24^-/low ^populations of SUM149**. **(A) **Number of CD44^high ^cells (per view field, ×10 magnification) of SUM149 after kinase silencing by siRNA at 5 n*M *for 72 hours. C, control without siRNA; Sc, scramble siRNA. *PLK1. **(B) **Growth inhibition of the sorted CD44^high^/CD24^-/low ^population of SUM149 by siRNA silencing of different kinases at 5 n*M *for 72 hours. Data are presented as mean ± SD of two independent tests. Kinases in the figure: *CDC2L1*, cell-division cycle 2-like 1 (PITSLRE proteins); *CHEK1*, CHK1 checkpoint homologue (*S. pombe*); *CDC2*, cell-division cycle 2, G_1 _to S and G_2 _to M; *CSNK2A2*, casein kinase 2, alpha prime polypeptide; *DGUOK*, deoxyguanosine kinase; *GCK*, glucokinase (hexokinase 4, maturity-onset diabetes of the young 2); *IRAK1*, interleukin-1 receptor-associated kinase 1; *MAP3K4*, alpha mitogen-activated protein kinase kinase kinase 4; *PDGFRA*, platelet-derived growth factor receptor, alpha polypeptide; *PFKFB2*, 6-phosphofructo-2-kinase/fructose-2,6-biphosphatase 2; *PIK3C2G*, phosphoinositide-3-kinase, class 2, gamma polypeptide; *PIK4CB*, phosphatidylinositol 4-kinase, catalytic, beta polypeptide; *PHKG2*, phosphorylase kinase, gamma 2 (testis); *PFTK1*, PFTAIRE protein kinase 1; *PLAU*, plasminogen activator, urokinase; *PLK1*, polo-like kinase 1 (*Drosophila*); *PRKCD*, protein kinase C, delta; *SKP2*: *S*-phase kinase-associated protein 2 (p45); *RPS6KA2*, ribosomal protein S6 kinase, 90 kDa, polypeptide 2; *TTK*, TTK protein kinase; *VRK2*, vaccinia-related kinase 2; *IHPK1*, inositol hexaphosphate kinase 1; *PKIG*, protein kinase (cAMP-dependent, catalytic) inhibitor gamma; *MAPK8IP3*, mitogen-activated protein kinase 8-interacting protein 3; *PRKD2*, protein kinase D2; *PI4K2B*, phosphatidylinositol 4-kinase type-II beta; *UCK1*, uridine-cytidine kinase 1; *LOC392265*, similar to cell-division protein kinase 5 (tau protein kinase II catalytic subunit) (TPKII catalytic subunit) (serine/threonine-protein kinase PSSALRE).

SUM149 cells consist of about 5% CD44^high ^cells under normal culture conditions. Of the 28 kinases tested, about half of them significantly reduced the number of CD44^high ^in the surviving population of SUM149 after siRNA treatments compared with the control (Figure 2A). In particular, 12 kinases, *CSNK2A2, GCK, MAP3K4, PDGFRA, PIK3C2G, PLAU, PLK1, SKP2, RPS6KA2, IHPK1, MAPK8IP3*, and *UCK1*, are the most active ones. It is noted also that deoxyguanosine kinase (*DGUOK*), conversely, significantly induced CD44^high ^cells after siRNA silencing.

When these 12 kinases were tested directly on TICs of sorted CD44^high^/CD24^-/low ^cells of SUM149 by silencing them with corresponding siRNAs at 5 n*M *for 72 hours, all of them, as expected, significantly inhibited the growth of the TICs compared with control (Figure 2B). The results confirmed our earlier observation of the reduced number of CD44^high ^cell in SUM149 after siRNA treatments of these 12 kinases (Figure 2A). *PLK1*, once again, had the most significant inhibitory effect on TICs.

### PLK1 is commonly expressed in breast cancer cells, and its expression is correlated positively to CD44

**Figure 3:**
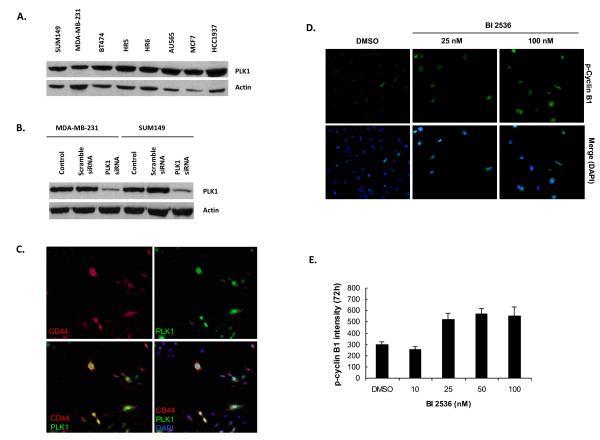
**PLK1 expression, its association with CD44, and its activity after BI 2536 inhibition**. **(A) **PLK1 expression in different breast cancer cells by immunoblotting. **(B) **PLK1 expression after *PLK1 *siRNA silencing in two TNBC (triple-negative breast cancer) cell lines, SUM149 and MDA-MB-231. **(C) **PLK1 is positively associated with CD44 expression with immunofluorescent assay. **(D) **Immunofluorescent images showing the accumulation of phospho-cyclin B1 in SUM149 cells after BI 2536 treatment (72 hours). DMSO, dimethyl sulfoxide; DAPI, 4ʹ,6-diamidino-2-phenylindole. **(E) **Quantitative analysis of phospho-cyclin B1 in SUM149 cells after BI 2536 treatment (72 hours).

Analysis with Western blot confirmed that PLK1 is commonly expressed in all eight breast cancer cell lines tested (Figure 3A). In particular, SUM149, MDA-MB-231, and HCC1937 are TNBC. Also, a siRNA silencing experiment confirmed the specific knockdown of *PLK1 *in both SUM149 and MDB-MB-231 cell lines (Figure 3B).

*PLK1 *is known to be highly associated with cell proliferation [28,31]. We therefore addressed whether it resides within the CD44^high ^subpopulation. By immunofluorescence, PLK1 was positively correlated to the expression of CD44, in that most (89% ± 14%) of CD44^high ^cells were also PLK1^high^, whereas the CD44^low ^cells failed to express high levels of PLK1 (Figure 3C). The high PLK1 in CD44^high ^cells may help maintain TICs and the ongoing proliferation of the tumor-initiating population. The results could partially explain our observation that the CD44^high ^subpopulation of SUM149 grew faster than did CD44^-/low ^cells (unpublished data).

### BI 2536 inhibited PLK1 activity, which led to the accumulation of phospho-cyclin B1 in SUM149 cells

Both qualitative and quantitative studies showed that PLK1 inhibition by BI 2536 at 25 n*M *or higher concentrations led to aberrant accumulation of phospho-cyclin B1 in the nuclear area of SUM149 cells (Figure 3D and 3E). The significant accumulation started 24 hours after treatment with 100 n*M *but not 10 n*M *BI 2536 (data not shown).

### PLK1 small-molecule inhibitor BI 2536 is as active as PLK1 siRNA against different breast cancer cell lines and TICs and induces apoptosis

**Figure 4:**
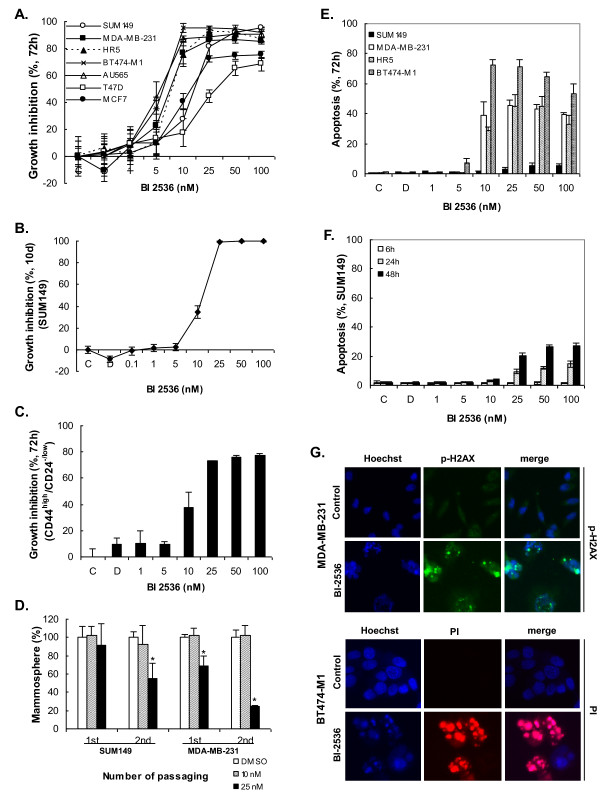
**Effect of PLK1 inhibition on breast cancer cell growth, apoptosis, and TICs**. **(A) **Percentage of growth inhibition of different breast cancer cells by a PLK1 small-molecule inhibitor BI 2536 at different concentrations for 72 hours. C, control (medium only). D, dimethyl sulfoxide only. **(B) **Percentage of growth inhibition of SUM149 cells by BI 2536 at different concentrations for 10 days. **(C) **Percentage of growth inhibition of sorted CD44^high^/CD24^-/low ^of SUM149 by BI 2536 for 72 hours. **(D) **Percentage of mammosphere reduction in SUM149 and MDA-MB-231 by BI 2536. *Significant difference from the control (Student *t *test, *P *< 0.05). **(E) **Percentage of apoptosis of different breast cancer cells caused by BI 2536 after 72 hours. **(F) **Time-course apoptosis of SUM149 treated with BI 2536 for 6, 24, and 48 hours. **(G) **Apoptotic images of the cells after BI 2536 treatment, as revealed by HCS (high-content screening) instrument (×10) with phospho-H2AX antibody or PI (propidium iodide) intake. Data are presented as mean ± SD of two independent tests. TICs, tumor-initiating cells.

Like its siRNA counterpart, PLK1 small-molecule inhibitor BI 2536 showed a significant growth-inhibitory effect on the cells of the seven different breast cancer cell lines under experimental conditions (Figure 4A). The active concentrations are as low as 1 to 5 n*M *with 80% to 90% growth reduction at 10 to 25 n*M *for most cancer cell lines after a 72-hour treatment. In particular, HR5, a trastuzumab-resistant cell line, is similarly sensitive to BI 2536 as is BT474-M1. A longer period of treatment of SUM149 cells with BI 2536 killed almost all cells at concentrations of 25 n*M *or higher (Figure 4B). More important, treatment with BI 2536 significantly inhibited the growth of sorted TICs of SUM149 compared with control (Figure 4C), further supporting its potential application in breast cancer.

In mammosphere assays on both SUM149 and MDA-MB-231 cells, BI 2536 treatment led to significant reduction of the sphere formation (Figure 4D). The results further confirmed our earlier observation of the inhibitory effect of BI 2536 on TICs on monolayer models.

Similar to *PLK1 *siRNA, BI 2536 also caused significant apoptosis at 10 to 100 n*M *in all four cell lines tested, a characteristic for PLK1 inhibition (Figure 4E through G). The loss of PLK1 activity triggered apoptosis in up to 70% of BT474-M1 cells that remained at the end of the 72-hour treatment. SUM149 had relatively fewer cells left at the end point and also fewer apoptotic cells compared with the other three cell lines, probably because the mass apoptosis occurred earlier. This was confirmed by time-course experiments at earlier times (Figure 4F), in which apoptosis peaked at about 48 hours after BI 2536 treatments. Together, the results from nuclear morphology (nuclear fragmentation and/or condensation), phospho-H2AX detection (an earlier indicator of apoptosis), and PI uptake (a late apoptosis indicator) clearly demonstrated the apoptosis in breast cancer cells caused by BI 2536.

**Figure 5:**
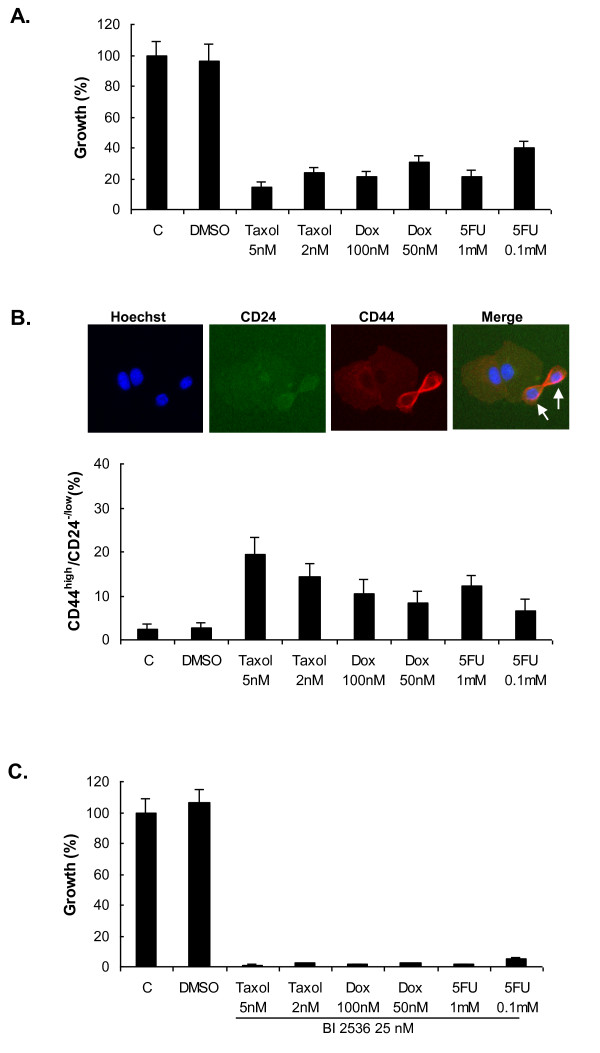
**Effect of combined treatment of Taxol, Dox, or 5FU followed by BI 2536 on SUM149 cells**. **(A) **Taxol, Dox, and 5FU inhibited the growth of SUM149 after 72 hours. C, control (medium only). **(B) **Drug treatment led to a higher percentage of CD44^high^/CD24^-/low ^TIC (tumor-initiating cell) subpopulation in surviving SUM149 cells after 72 hours. Top image panel: CD44^high^/CD24^-/low ^cells (arrows) after 5FU treatment (0.1 m*M*, 72 hours) as viewed by HCS (high-content screening) instrument (×10). **(C) **Combined treatment with the drugs (Taxol, Dox, or 5FU) for 72 hours, followed by BI 2536 for another 96 hours, significantly reduced cell growth, even though the drugs induced a higher percentage of the CD44^high^/CD24^-/low ^subpopulation. All data are presented as mean ± SD of two independent tests. DMSO, dimethyl sulfoxide; Taxol, paclitaxel; Dox, doxorubicin; 5FU, 5-fluorouracil.

An unfortunate consequence of chemotherapies used to treat breast cancer is that they induce TICs [15,17]. Here we show that Taxol, Dox, and 5FU inhibited cancer cell growth, while at the same time, they induced a higher proportion of CD44^high^/CD24^-/low ^cells from about 2% in the controls to about 6% to 20% in the surviving populations after a 72-hour exposure (Figure 5A and 5B). After the induction of CD44^high^/CD24^-/low ^cells by these drugs, we subsequently exposed the cells to BI 2536 for an additional 4 days. The sequential treatment led to almost complete cell death (Figure 5C). This demonstrates that although resistant cells exist after the drug treatments, they remain sensitive to BI 2536 at low concentration. Most important, BI 2536 can be used to overcome chemotherapy-induced TICs and suggested a potential to prevent relapse.

## Discussion

The key functions of kinases in signal transduction for all organisms make them very attractive targets for therapeutic interventions in many diseases, including cancers [18,21,23]. Several kinase inhibitors have been used for the treatment of cancer, such as imatinib, gefitinib, erlotinib, fasudil, and rapamycin [21,22]. Genome-wide gene-library screens have proved an excellent tool in identifying such biologic targets [18-20,33]. In this study, we screened a human kinase siRNA library against a TNBC cell line, SUM149, for *in vitro *growth inhibition. As a result, 85 kinases, including *PLK1*, were identified to be strongly inhibitory against the cell growth once they were silenced by corresponding siRNAs. The diverse functional groups of the kinases identified in this study demonstrate their important roles in regulating the growth of breast cancer cells. In particular, about one fourth of the identified kinases were previously proposed to be the targets or already are in clinical trials for breast cancers (Additional file 1). *AURKB, BUB1B, CHEK1, EPHB6, GSK3, MAPK*s, *MYLK, NEK*s, *PDGFRA, PLAU, PLK1, PKC, RSK, SKP2*, and *TTK *are just a few of them [21,22,34-38]. Kinases *BUB1, CHEK1, IRAK1, TTK, RYK*, and *VRK2*, identified in this study, for example, have been reported to be highly overexpressed in ER-negative breast tumors and were critical for the growth of either ER-negative only or both ER-positive and -negative breast cancer cells [9,23]. These studies validate our approach of a genome-wide gene library screen in target discovery for TNBC. In addition, most of the 28 active kinases that were selected for further study showed a broad spectrum of activity, not only on TNBC, but also on other ER/HER2-positive breast cancer groups. Thus our study provides a broad basis of potential therapeutic targets, not only to TNBC, but also to other subtypes of breast cancers.

Cancer relapse has long been a clinical problem in breast cancer treatment. Recent theories and evidence have pointed to cancer stem cells or TICs for the root cause. The cancer stem cell hypothesis proposed that tumors are driven by a cellular component that retains stem cell properties, including self-renewal, tumorigenicity, and multilineage differentiation capacity [11,12]. In breast cancer, several subpopulations, such as CD44^high^/CD24^-/low^, CD133/PROM/prominin, and ALDEFLUOR^+^, have been shown to contain highly enriched cancer stem cells [10,15,39]. Targeting such a subpopulation, as well as the bulk cancer population, could lead to complete cure of the cancer diseases. In this study, after identifying the active kinases, we questioned whether any of these kinases had also played a role in TICs. When we analyzed the CD44^high ^population in the surviving cells after siRNA treatment, 12 of these 28 selected kinases significantly reduced the population of CD44^high ^cells. This led to the test of these 12 kinases directly against a sorted CD44^high^/CD24^-/low ^subpopulation of SUM149. As expected, they inhibited the growth of the sorted TICs. The confirmation of the anti-TIC subpopulation is particularly significant, given the accepted role of TICs in drug resistance and cancer relapse. The involvement of kinases in TICs of different cancers has been reported [16,25-27], and our study provides new evidence for further exploration on these kinases and TICs, in particular, for better breast cancer therapy.

*PLK1 *is one of the four mammalian *PLK *family members. Its prime role in mammalian cells is the control of mitotic progression, particularly the regulation of proteins that are involved in metaphase-anaphase transition and mitotic exit. The activity and concentration of this kinase are crucial for the precise regulation of cell division [40]. PLK1 was reported to be overexpressed in a broad spectrum of cancer types, and its expression often correlates with poor patient prognosis [19,40,41]. PLK1 has long been established as a marker for cellular proliferation [42]. Its levels in non-small-cell lung cancer tumors correlate inversely with survival, indicating that PLK1 may have prognostic value [43]. This was later confirmed in multiple cancer types [40]. PLK1 expression has also been shown to be a reliable marker for identifying a high risk of metastasis in malignant melanomas [44]. In a cluster analysis of 82 normal and malignant breast specimens with cDNA array, *PLK1 *was found overexpressed to various extents in a subgroup of patient tumors, designated class A, which contains a higher proportion of patients with metastases and a greater risk of recurrence [45,46]. Given this, it would be important to evaluate the potential for *PLK1 *inhibitors in patients with metastatic disease as a future direction. Numerous studies have now established that *PLK1 *is a prime target for drug development in proliferative diseases such as breast cancer [37,40]. Inhibition of *PLK1 *leads to mitotic arrest, interruption of cytokinesis, and apoptosis in susceptible tumor cell populations.

In this study, the expression of *PLK1 *in different breast cancer subtypes was confirmed, and its inhibition led to growth inhibition and apoptosis on all breast cancer cell lines tested, indicating a broad application in breast cancer treatment. The sensitivity to *PLK1 *depletion has been linked to *p53 *status in cancer cells, although conflicting reports exist [19,40]. In this study, AU565 (ER^- ^and HER2^+^), which has a wild-type *p53*, is equally sensitive to PLK1 inhibition as MDA-MB-231 (TNBC), which is *p53 *mutant. Similarly, of the three slightly less-sensitive cell lines, SUM149 (TNBC) is *p53 *mutant, whereas MCF7 and T47D (ER^+ ^and HER2^-^) are both *p53 *wt. The results indicate that sensitivity to PLK1 inhibition may not be linked directly to *p53 *status. Although a normal cell line was not included in the study for comparison, numerous studies, both *in vivo *and even clinical trials, have established that *PLK1 *inhibition by siRNA or BI 2536 is well tolerated, with neutropenia being the main side effect [26,28,39,47-49]. PLK1 inhibitors seem also to have an advantage over mitotic inhibitors such as the taxanes or vinca alkaloids, because they do not induce the neurotoxicity, as do these earlier inhibitors [50,51]. Combination of *PLK1 *siRNA with chemotherapeutic drugs also enhanced the sensitivity toward Taxol and trastuzumab (Herceptin) in a synergistic manner [32]. Most important, our study represents the first attempt to associate *PLK1 *with TICs in breast cancer. Of the 28 selected kinases in our focused studies, *PLK1 *is the leading candidate, based on its activity in inhibiting cancer cell growth, and in particular, its activity against the TICs once silenced by siRNA or by the small-molecule inhibitor, BI 2536. Fillmore and Kuperwasser [15] reported that current chemotherapeutic agents for breast cancer, such as Taxol and 5FU, actually induced TICs. This is indeed the case for Taxol, Dox, and 5FU, under our test conditions. In addition, when these drug treatments were followed with BI 2536, few cells survived, even though they induced CD44^high^/CD24^-/low ^cells under the experimental conditions. Interestingly, Gleixner *et al. *[52] recently reported that inhibiting PLK1 with BI 2536 could override imatinib resistance in chronic myeloid leukemia. Whether this is related to the activity of PLK1 on TICs of the disease remains to be explored.

Although *PLK1 *is the focus of our study for its significant growth inhibition on breast cancer, availability of small-molecular inhibitors, and the safety data in clinical trials of different cancer treatment [28,40,49], several other active kinases identified in this study deserve further study for their roles in TICs in breast cancer, such as *SKP2 *and *PLAU *(*uPA*), which inhibited the growth of sorted CD44^high^/CD24^-/low ^cells of SUM149. Indeed, uPA/PAI-1 is the only biomarker to have been conferred with LOE-1 as a definitive prognostic marker of poor disease outcome in early breast cancer [53]. Furthermore, the guidelines of the American Society of Clinical Oncology also consider the components of the uPAS to be promising targets for future therapeutic studies [53]. The first inhibitors of uPA have now been tested in oncology trials worldwide, and one of the compounds, WX-671, has received US FDA approval for a phase II trial in metastatic breast cancer in combination with chemotherapy [53,54]. Evidence exists that uPA is highly expressed in CD44^+ ^cells [55]. Conceptually, this fits with the idea that TICs are invasive [12], and as such, they are found in circulating tumor cells from patients [56]. High levels of uPA are also associated with breast cancer relapse, which again could underpin the idea that its expression in TICs is associated with drug resistance. SKP2 is overexpressed in a subset of breast carcinomas (ER^- ^and HER2^-^) and might play a role in the development of resistance to anti-estrogens [34]. Overexpression of SKP2 is associated with resistance to preoperative doxorubicin-based chemotherapy in primary breast cancer [36]. Further confirmation of this effect on TICs could help define better therapeutic strategies. It should be noted also that our primary screen targets the overall growth inhibition of SUM149 rather than the TICs; it is possible that some kinases could be missed from the hit list if they are active only on the TICs, but not or weakly active on the bulk of the cancer cell population.

## Conclusions

The inhibition of PLK1 led to significant growth inhibition, either alone or in combination with other drugs, on different breast cancer cells and TICs, making them promising therapeutic targets in the treatment of TNBC and other breast cancers.

## Abbreviations

5FU: 5-fluorouracil; DAPI: 4ʹ,6-diamidino-2-phenylindole; DMEM: Dulbecco Modified Eagle medium; DMSO: dimethyl sulfoxide; Dox: doxorubicin; ER: estrogen receptor; FBS: fetal bovine serum; HCS: high content screening; HER2: human epidermal growth factor 2; PBS: phosphate-buffered solution; PI: propidium iodide; siRNA: small interfering RNA; Taxol: paclitaxel; TICs: tumor-initiating cells; TNBC: triple-negative breast cancer.

## Competing interests

The authors declare that they have no competing interests.

## Authors' contributions

KH performed siRNA screens, growth assays, cyclin B1 assays, apoptosis measurements, and prepared the manuscript. JL performed mammosphere formation and immunoblotting assays. AF performed immunofluorescence of PLK1 and cyclin B1. SED conceived the study, arranged research funding, and prepared the manuscript. All authors read and approved the final manuscript.

## Supplementary Material

Additional file 1 **Complete list of active kinases identified in siRNA library screen**. Table 1. Active kinases identified in the siRNA library screen. The table lists the accession numbers, symbols, and brief description of the kinases identified in the library screen as well as the growth inhibition (percentage) of the kinases after siRNA silencing at 5 n*M *for 72 hours under the test conditions. Click here for file
