# Supplementary material for: Small interfering RNA library screen identified polo-like kinase-1 (PLK1) as a potential therapeutic target for breast cancer that uniquely eliminates tumor-initiating cells
Source: Breast Cancer Res. 2012 Feb 6;14(1):R22. doi: 10.1186/bcr3107 (PMC3496140; doi:10.1186/bcr3107)
Supplement: Additional file 1 — Complete list of active kinases identified in siRNA library screen. Table 1. Active kinases identified in the siRNA library screen. The table lists the accession numbers, symbols, and brief description of the kinases identified in the library screen as well as the growth inhibition (percentage) of the kinases after siRNA silencing at 5 nM for 72 hours under the test conditions. [file bcr3107-S1.DOC]

Additional file 1: Complete list of active kinases identified in siRNA library screen

**Table 1. Active kinases identified in the siRNA library screen**

| Accession No. | Symbol | Description | Growth inhibition (%)  Sequence C | Growth inhibition (%)  Sequence D |
| --- | --- | --- | --- | --- |
| Cell cycle/division | | | | |
| NM-033487 | CDC2L1 | cell division cycle 2-like 1 (PITSLRE proteins) | 67.2 | 74.5* |
| NM-001261 | CDK9 | cyclin-dependent kinase 9 (CDC2-related kinase) | 35.5 | 51.4 |
| NM-001274 | CHEK1 | CHK1 checkpoint homolog (S. pombe) | 65.7 | 56.1 |
| NM-001826 | CKS1B | CDC28 protein kinase regulatory subunit 1B | 52.5 | 52.5 |
| NM-001786 | CDC2 | cell division cycle 2, G1 to S and G2 to M | 65.5* | 70.5* |
| NM-005983 | SKP2 | S-phase kinase-associated protein 2 (p45) | 62.7 | 58.7 |
| XM-498294 | LOC392265 | similar to Cell division protein kinase 5 (Tau protein kinase II catalytic subunit) (TPKII catalytic subunit) (Serine/threonine-protein kinase PSSALRE) | 69.5 | 65.4* |
| MAP kinase | | | | |
| NM-005922 | MAP3K4 | mitogen-activated protein kinase kinase kinase 4 | 59.3 | 55.6 |
| NM-002756 | MAP2K3 | mitogen-activated protein kinase kinase 3 | 59.8 | 47.1 |
| NM-003954 | MAP3K14 | mitogen-activated protein kinase kinase kinase 14 | 35.3 | 42.9 |
| NM-005456 | MAPK8IP1 | mitogen-activated protein kinase 8 interacting protein 1 | 70.2 | 45.1 |
| NM-006116 | MAP3K7IP1 | mitogen-activated protein kinase kinase kinase 7 interacting protein 1 | 43.5* | 54.8 |
| NM-015133 | MAPK8IP3 | mitogen-activated protein kinase 8 interacting protein 3 | 56.0* | 56.0 |
| NM-024117 | MAPKAP1 | mitogen-activated protein kinase associated protein 1 | 52.8 | 40.7 |
| PIK | | | | |
| NM-005027 | PIK3R2 | phosphoinositide-3-kinase, regulatory subunit 2 (p85 beta) | 53.0 | 68.1 |
| NM-002651 | PIK4CB | phosphatidylinositol 4-kinase, catalytic, beta polypeptide | 47.5 | 65.3 |
| NM-006219 | PIK3CB | phosphoinositide-3-kinase, catalytic, beta polypeptide | 31.3 | 53.2* |
| NM-018323 | PI4K2B | phosphatidylinositol 4-kinase type-II beta | 66.4* | 34.6* |
| NM-004570 | PIK3C2G | phosphoinositide-3-kinase, class 2, gamma polypeptide | 74.0* | 63.9* |
| Protein kinase | | | | |
| NM-006254 | PRKCD | protein kinase C, delta | 57.5 | 61.3* |
| NM-002760 | PRKY | protein kinase, Y-linked | 42.9 | 39.4 |
| NM-003137 | SRPK1 | SFRS protein kinase 1 | 49.5* | 48.7 |
| NM-002740 | PRKCI | protein kinase C, iota | 37.9 | 41.8 |
| NM-002737 | PRKCA | protein kinase C, alpha | 38.7 | 46.5 |
| NM-021135 | RPS6KA2 | ribosomal protein S6 kinase, 90kDa, polypeptide 2 | 66.6* | 33.1 |
| NM-003318 | TTK | TTK protein kinase | 61.6 | 66.1* |
| NM-181805 | PKIG | protein kinase (cAMP-dependent, catalytic) inhibitor gamma | 63.3 | 67.6* |
| NM-007229 | PACSIN2 | protein kinase C and casein kinase substrate in neurons 2 | 35.8 | 51.4 |
| NM-016457 | PRKD2 | protein kinase D2 | 57.3 | 63.2 |
| NM-153335 | LYK5 | protein kinase LYK5 | 56.1* | 39.3 |
| NM-012395 | PFTK1 | PFTAIRE protein kinase 1 | 70.6 | 35.9* |
| Miscellaneous | | | | |
| NM-001619 | ADRBK1 | adrenergic, beta, receptor kinase 1 | 49.1 | 51.9 |
| NM-001204 | BMPR2 | bone morphogenetic protein receptor, type II (serine/threonine kinase) | 39.0 | 55.6 |
| NM-001743 | CALM2 | calmodulin 2 (phosphorylase kinase, delta) | 40.2 | 57.7* |
| NM-001211 | BUB1B | BUB1 budding uninhibited by benzimidazoles 1 homolog beta (yeast) | 58.7* | 39.0* |
| NM-001896 | CSNK2A2 | casein kinase 2, alpha prime polypeptide | 67.3* | 57.1* |
| NM-001929 | DGUOK | deoxyguanosine kinase | 55.6* | 66.3* |
| NM-000162 | GCK | glucokinase (hexokinase 4, maturity onset diabetes of the young 2) | 74.5* | 48.8 |
| NM-002093 | GSK3B | glycogen synthase kinase 3 beta | 55.7 | 45.3 |
| NM-001569 | IRAK1 | interleukin-1 receptor-associated kinase 1 | 74.0* | 44.6 |
| NM-004445 | EPHB6 | EPH receptor B6 | 49.0* | 47.8* |
| NM-001982 | ERBB3 | v-erb-b2 erythroblastic leukemia viral oncogene homolog 3 (avian) | 53.6 | 49.2* |
| NM-005308 | GRK5 | G protein-coupled receptor kinase 5 | 53.6* | 51.6* |
| NM-014776 | GIT2 | G protein-coupled receptor kinase interactor 2 | 48.9* | 49.0 |
| NM-001556 | IKBKB | inhibitor of kappa light polypeptide gene enhancer in B-cells, kinase beta | 41.6 | 59.9* |
| NM-005592 | MUSK | muscle, skeletal, receptor tyrosine kinase | 52.4 | 53.2 |
| NM-002513 | NME3 | non-metastatic cells 3, protein expressed in | 54.9 | 63.6* |
| NM-006206 | PDGFRA | platelet-derived growth factor receptor, alpha polypeptide | 69.8 | 56.2 |
| NM-006212 | PFKFB2 | 6-phosphofructo-2-kinase/fructose-2,6-biphosphatase 2 | 56.9 | 62.2 |
| NM-138733 | PGK2 | phosphoglycerate kinase 2 | 61.5 | 47.0 |
| NM-002637 | PHKA1 | phosphorylase kinase, alpha 1 (muscle) | 54.8 | 51.2 |
| NM-000292 | PHKA2 | phosphorylase kinase, alpha 2 (liver) | 58.4 | 39.7 |
| NM-000294 | PHKG2 | phosphorylase kinase, gamma 2 (testis) | 76.0* | 54.2 |
| NM-002626 | PFKL | phosphofructokinase, liver | 48.6 | 49.5* |
| NM-002627 | PFKP | phosphofructokinase, platelet | 46.6 | 42.8 |
| NM-002658 | PLAU | plasminogen activator, urokinase | 61.1 | 52.4 |
| NM-005030 | PLK1 | polo-like kinase 1 (Drosophila) | 90.3* | 82.5* |
| NM-002958 | RYK | RYK receptor-like tyrosine kinase | 40.8 | 45.4* |
| NM-003600 | STK6 | serine/threonine kinase 6 | 44.2 | 51.2 |
| NM-003957 | STK29 | serine/threonine kinase 29 | 35.6 | 37.7 |
| NM-006374 | STK25 | serine/threonine kinase 25 (STE20 homolog, yeast) | 41.4 | 40.6* |
| NM-032430 | BRSK1 | BR serine/threonine kinase 1 | 40.8 | 39.9 |
| NM-000459 | TEK | TEK tyrosine kinase, endothelial (venous malformations, multiple cutaneous and mucosal) | 43.6 | 58.1 |
| NM-003886 | AKAP4 | A kinase (PRKA) anchor protein 4 | 38.2 | 35.5 |
| NM-006422 | AKAP3 | A kinase (PRKA) anchor protein 3 | 46.3 | 41.5 |
| NM-006296 | VRK2 | vaccinia related kinase 2 | 53.8 | 56.5 |
| NM-003384 | VRK1 | vaccinia related kinase 1 | 34.3 | 46.2 |
| NM-153273 | IHPK1 | inositol hexaphosphate kinase 1 | 57.0 | 53.3 |
| NM-005876 | APEG1 | aortic preferentially expressed protein 1 | 74.1 | 44.9 |
| NM-014397 | NEK6 | NIMA (never in mitosis gene a)-related kinase 6 | 48.0 | 47.2 |
| NM-033116 | NEK9 | NIMA (never in mitosis gene a)- related kinase 9 | 42.1 | 49.2 |
| XM-292160 | MGC75495 | similar to Serine/threonine-protein kinase Nek1 (NimA-related protein kinase 1) | 58.3 | 48.4 |
| NM-004783 | TAOK2 | TAO kinase 2 | 50.3 | 42.6 |
| NM-004217 | AURKB | aurora kinase B | 51.2 | 47.8* |
| AB014541 | AATK | apoptosis-associated tyrosine kinase | 36.6 | 35.2 |
| NM-014978 | SORCS3 | sortilin-related VPS10 domain containing receptor 3 | 45.8 | 48.7* |
| NM-022445 | TPK1 | thiamin pyrophosphokinase 1 | 47.3 | 36.4 |
| NM-013254 | TBK1 | TANK-binding kinase 1 | 41.8 | 41.3 |
| NM-020240 | CDC42SE2 | CDC42 small effector 2 | 57.7 | 51.4 |
| NM-020836 | KIAA1446 | brain-enriched guanylate kinase-associated protein | 48.0 | 39.4 |
| NM-025144 | ALPK1 | alpha-kinase 1 | 57.9 | 42.6 |
| NM-031432 | UCK1 | uridine-cytidine kinase 1 | 68.6* | 56.7 |
| NM-033118 | MYLK2 | myosin light chain kinase 2, skeletal muscle | 49.7 | 64.9* |
| NM-152835 | PDIK1L | PDLIM1 interacting kinase 1 like | 48.8 | 36.4 |
| NM-152619 | DCAMKL2 | doublecortin and CaM kinase-like 2 | 34.0 | 39.5 |

*Apoptosis is at least 5% more than the control based on nuclear properties (morphology and higher Hoechst intensity).
